# Peroxiredoxins in Neurodegenerative Diseases

**DOI:** 10.3390/antiox9121203

**Published:** 2020-11-30

**Authors:** Monika Szeliga

**Affiliations:** Mossakowski Medical Research Centre, Department of Neurotoxicology, Polish Academy of Sciences, 5 Pawinskiego Street, 02-106 Warsaw, Poland; mszeliga@imdik.pan.pl; Tel.: +48-(22)-6086416

**Keywords:** peroxiredoxin (PRDX), oxidative stress, nitrosative stress, neurodegenerative disease

## Abstract

Substantial evidence indicates that oxidative/nitrosative stress contributes to the neurodegenerative diseases. Peroxiredoxins (PRDXs) are one of the enzymatic antioxidant mechanisms neutralizing reactive oxygen/nitrogen species. Since mammalian PRDXs were identified 30 years ago, their significance was long overshadowed by the other well-studied ROS/RNS defense systems. An increasing number of studies suggests that these enzymes may be involved in the neurodegenerative process. This article reviews the current knowledge on the expression and putative roles of PRDXs in neurodegenerative disorders such as Alzheimer’s disease, Parkinson’s disease and dementia with Lewy bodies, multiple sclerosis, amyotrophic lateral sclerosis and Huntington’s disease.

## 1. Introduction

Under physiological conditions, reactive oxygen species (ROS, e.g., superoxide anion, O2·-; hydrogen peroxide, H_2_O_2_; hydroxyl radical, ·OH; organic hydroperoxide, ROOH) and reactive nitrogen species (RNS, e.g., nitric oxide, NO·; peroxynitrite, ONOO-) are constantly produced as a result of normal cellular metabolism and play a crucial role in signal transduction, enzyme activation, gene expression, and regulation of immune response [1]. The cells are endowed with several enzymatic (e.g., glutathione peroxidase (GPx); peroxiredoxin (PRDX); thioredoxin (TRX); catalase (CAT); superoxide dismutase (SOD)), and non-enzymatic (e.g., glutathione (GSH); quinones; flavonoids) antioxidant systems that minimize the levels of ROS and RNS. When the balance between cellular oxidants and antioxidants is shifted in favor of the oxidants, the cells become subject of oxidative/nitrosative stress, a deleterious process that damages DNA, RNA, proteins and lipids. Oxidative/nitrosative stress perturbs cell health and viability and triggers a variety of cellular responses via generation of secondary reactive species which in turn leads to cell death [2]. Several exogenous (e.g., chemicals, ultraviolet, X and γ rays) and endogenous (e.g., metabolic and inflammatory processes, cell signaling) agents can induce oxidative damage.

The central nervous system (CNS) is particularly vulnerable to oxidative stress because of its high oxygen demand and high concentrations of easily peroxidizable lipids [3,4]. An increasing evidence indicates a link between oxidative stress and certain pathologies, including neurodegenerative diseases which are characterized by progressive degeneration and death of neurons leading to motor and cognitive dysfunction (for exhaustive reviews [5,6]). Of note, the contribution of astrocytes and microglia to neurodegeneration has also been postulated [7,8]. Whether an elevated level of ROS/RNS observed in the brain of patients with neurodegenerative diseases is a cause or a consequence of the neurodegenerative process remains unclear. Recently, the role of deregulated expression or/and activity of the antioxidant enzymes in neurodegeneration has received considerable attention. Since mammalian PRDXs were identified approximately 30 years ago, their significance was long overshadowed by the other well-studied ROS/RNS defense mechanisms. In the past two decades, several studies demonstrated altered levels of the particular PRDX proteins in the brain of patients with distinct neurodegenerative diseases. The significance of these alterations remains a matter of discussion.

## 2. PRDXs: A General Overview

PRDXs (EC: 1.11.1.15) are a family of thiol-dependent peroxidases catalyzing the reduction of hydrogen peroxide, peroxynitrite and alkyl hydroperoxides [9]. All PRDXs contain a conserved active site cysteine, named the peroxidatic cysteine (CP), which reacts with peroxide to form a cysteine sulfenic acid (R-SOH) and releases water or the corresponding alcohol. Some of the PRDXs contain also a second cysteine, named the resolving cysteine (CR), which attacks the R-SOH to form a water molecule and a disulfide bond in the protein. Human cells express six PRDX isoforms, which can be divided into three subgroups:

(1) Typical 2-Cys PRDXs containing both the peroxidatic and resolving Cys residues and require both of them for catalytic function; this subgroup includes PRDX1-4;
(2) Atypical 2-Cys PRDXs, which contain only the peroxidatic Cys, but require one additional Cys residue for catalytic activity; this subgroup includes PRDX5;
(3) 1-Cys PRDXs, which contain a single redox-active peroxidatic Cys residue in the N-terminus; this subgroup includes PRDX6 [9].

The typical 2-Cys PRDXs are obligate homodimers containing two identical active sites. In the first step of the peroxidase reaction, CP attacks the peroxide substrate and is oxidized to a cysteine sulfenic acid. In the second step of the reaction, a cysteine sulfenic acid from one subunit is attacked by CR located in the C terminus of the other subunit which results in the formation of an intersubunit disulfide bond. The catalytic cycle is completed by the reduction of the disulfide bond by one of several oxidoreductases, such as TRX, tryparedoxin, alkyl hydroperoxide reductase. The atypical 2-Cys PRDXs are monomers with both CP and CR located in the same molecule, therefore the condensation reaction results in the formation of an intramolecular disulfide bond. To reduce the disulfide, this class of PRDXs uses oxidoreductase, most likely TRX, as an electron donor. The detailed mechanism of action of the 1-Cys PRDXs has not yet been fully clarified, but presumably one donor thiol forms a transient mixed disulfide bond with the enzyme, followed by its reduction by a second donor thiol. PRDX6, the only member of 1-Cys PRDX subgroup, uses as a reductant glutathione, ascorbate, lipoic acid or cyclophilin, but not TRX [10,11].

**Figure 1:**
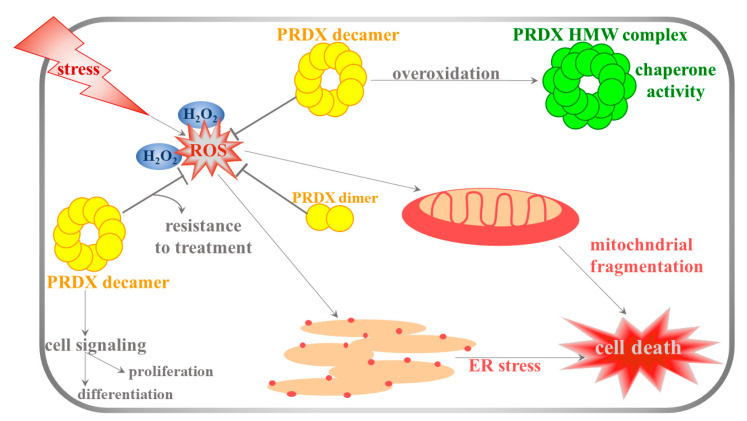
Functions of proxiredoxins (PRDXs) in the cell. Increased level of ROS triggers mitochondrial fragmentation and endoplasmic reticulum (ER) stress which leads to cell death. PRDXs protect cells by ROS scavenging. Most PRDXs form decamers or, upon overoxidation, high-molecular-weight (HMW) complexes which gain chaperone activity. In cancer cells, high levels of PRDXs contribute to resistance to treatment. Apart from ROS scavenging activity, PRDXs may be implicated in cell signaling, differentiation and proliferation.

In the reduced state, the typical 2-Cys PRDXs form decamers (PRDX1, 2, and 4) or dodecamers (PRDX3) [12,13]. Overoxidation results in the formation of high-molecular-weight (HMW) complexes, which gain molecular chaperone activity that protects cells against stress-induced protein unfolding [14,15]. Aside from peroxidase and chaperone activities, PRDXs interact with the other proteins, and therefore are involved in diverse intracellular processes such as neuronal differentiation, cell growth, apoptosis, carcinogenesis, and resistance of cancer cell to treatment (reviewed in [16]; Figure 1). Of note, PRDX6 contains a lipase motif (GXSXG), and an increasing number of studies points to the phospholipase A2 (PLA2) activity of this protein (reviewed in [17]).

The expression of the genes coding for PRDXs is regulated by several transcription factors as well as epigenetic events. The activity of PRDXs proteins is modulated by post-translational modifications including oxidation, phosphorylation, nitrosylation and proteolytic cleavage. The detailed analysis of both issues goes beyond the focus of this review which presents current knowledge regarding the role of the particular PRDX proteins in the neurodegenerative disorders. The literature search was conducted in PubMed electronic database updated until November, 2020. Non-English publications were excluded. The following keywords, either alone or in different combinations, were used: “peroxiredoxin”, “brain”, “neurodegenerative disease”, “Alzheimer’s disease”, “Parkinson’s disease”, dementia with Lewy bodies”, multiple sclerosis”, amyotrophic lateral sclerosis”, “Huntington’s disease”. Manual searching in the reference citations of identified articles has also been performed.

## 3. PRDXs in the CNS

The expression of PRDXs is cell type-specific in the CNS. Moreover, particular PRDXs localize in different cellular compartments. Hence, PRDX1 is primarily expressed in human astrocytes, the prominent expression is also observed in ependymocytes, while it is undetectable in human neurons [18]. In the mouse brain strong PRDX1 expression has been detected in oligodendrocytes and in microglia, while its traces or lack was observed in the neuronal populations [19,20,21]. In the rat brain the PRDX1 immunostaining was detected in oligodendrocytes, astrocytes and ependymocytes, and a few neuronal cells were immunopositive [22].

PRDX2 expression was documented both in human neurons [18] and astrocytes [23]. In the mouse brain, PRDX2 was found in neurons, mainly in cytoplasm, but could also be observed in the nucleus [19,20]. PRDX3 appears to be localized in neuronal mitochondria [19,20,24,25], although Godoy and co-workers registered PRDX3 staining mainly in glial cells [26]. The expression of PRDX4 has been documented in neurons and astrocytes [19], but also in oligodendrocytes [20]. While in neurons this protein was localized in the cytoplasm, in oligodendrocytes PRDX4 immunoreactivity was observed in the nuclei [21]. Similarly to PRDX3, PRDX5 has been detected in neuronal mitochondria and cytoplasm [19,20]. PRDX6 has been detected in human astrocytes and at low levels in neurons but not in microglia or oligodendrocytes [27]. In the mouse brain PRDX6 has been found mainly in astrocytes and some neurons [19], but its expression has also been noted in oligodendrocytes [20,26]. The presence of PRDX6 has likewise been detected in cerebrospinal fluid (CSF) [28]. Data on expression of particular PRDXs in the CNS are summarized in Table 1.

## 4. PRDXs in Neurodegenerative Diseases

### 4.1. PRDXs in Alzheimer’s Disease (AD)

AD is the most common type of dementia, accounting for 60−70% of 50 million people suffering from dementia worldwide [29]. It is a degenerative brain disease thought to begin 20 years before symptoms arise. Deterioration in learning and memory occurs due to damage of neurons in the parts of the brain involved in cognitive functions. In the course of disease progresses, neurons in the other parts of the brain become damaged or destroyed. Finally, the patient is unable to carry out basic bodily functions and requires around-the-clock care [30]. The etiology of AD is complex and still not fully clarified. The accumulation of amyloid β (Aβ) is a central feature of AD, although it is not clear whether Aβ accumulation alone is sufficient to produce symptoms. Abnormally hyperphosphorylated and aggregated Tau protein has been suggested as a facilitator of the downstream effects of the Ab accumulation. Genetic mutations in the genes coding for presenilin 1 (PS1), presenilin 2 (PS2), amyloid beta precursor protein (APP), and Trisomy 21 may also be involved in the pathogenesis of AD [30].

In the first study on PRDX1 expression, the level of this protein was significantly elevated in temporal cortex, occipital cortex and thalamus from patients with AD compared to controls and displayed a tendency to increase in the other brain regions [31]. However, a later study by the same group revealed lack of differences in the amount of PRDX1 in frontal cortex between AD and control tissues [32]. Notwithstanding, an increased level of PRDX1 has been documented in frontal cortex [33] and inferior parietal lobule [34] from patients with AD. Moreover, Cumming and co-workers provided an evidence that PRDX1 was upregulated in Aβ-resistant rat pheochromocytoma cells PC12 and overexpression of PRDX1 protected both PC12 cells and rat primary hippocampal neurons against Aβ toxicity [33]. A similar protective effect of PRDX1 transfection was observed in N2- differentiated SH-SY5Y human neuroblastoma cells treated with Aβ. Moreover, overexpression of PRDX1 diminished the level of pro-BDNF, an inductor of neuronal apoptosis significantly elevated upon Aβ challenge, and increased TrkB as well as pERK5 involved in neuronal survival [35]. These results indicate that PRDX1 is not only per se an antioxidant protein, but it also affects, most likely by modulating oxidative stress, signal transduction pathways implicated in neuroprotection and cell death.

A recent study has shown a colocalization of PRDX1, but also PRDX4, with p-AMPK, p-mTOR and p-tau in the brain tissues from patients with AD. Phosphorylation of AMPK and mTOR was detected in neurons, strongly indicating neuronal localization of PRDX1. Of note, the cells possessing a substantial amounts of p-AMPK, p-mTOR and p-tau displayed a significant reduction of PRDX1 and PRDX4 along with an increased level of mitochondrial DNA and cellular protein oxidation, suggesting that downregulation of antioxidant enzymes and oxidative stress are the early events triggering AMPK/mTOR activation in AD [36].

As already mentioned in Section 2, the activity of PRDX proteins may be regulated by several post-translational modifications. PRDX1 is a target of histone deacetylase 6 (HDAC6) and deacetylation of PRDX1 inhibits its reducing activity [37]. Therefore, upregulation of HDAC6 observed in the brains of AD patients [38] may diminish antioxidant activity of PRDX1. Indeed, PRDX1 acetylation was decreased in the brains of AD patients compared to control and it was regulated by both Aβ and HDAC6. Elevation of PRDX1 acetylation by HDAC6 inhibition rescued oxidative stress and mitochondrial transport in AD model mice suggesting that modulation of PRDX1 acetylation could be one of the therapeutic strategies for AD [39].

A significantly increased level of PRDX2 was observed in the thalamus from patients with AD compared to controls. A tendency to elevated level of this protein was also observed in the other regions of brains from patients with AD [31,32]. In a very recent proteomic study, PRDX2 appeared to be increased markedly in hippocampus of AD brains compared to controls [34]. Yao and co-workers has documented elevated level of PRDX2 in cerebral cortex of patients with AD and in transgenic mice expressing amyloid β binding alcohol dehydrogenase (ABAD) and a mutated form of the amyloid precursor protein (APP). Furthermore, overexpression of PRDX2 protected cortical neurons against Aβ induced toxicity indicating that an increased expression of this protein observed in the AD brains is a characteristic feature of neurons attempting to protect themselves in a high Aβ concentration environment [40].

An increased level of PRDXs is assumed to play a neuroprotective role. However, if the level of ROS exceeds the detoxifying abilities of the antioxidant system in the cell, PRDXs become oxidatively inactivated. Indeed, Cumming and co-workers identified an inactive version of PRDX2 with an oxidized catalytic cysteine residue in the AD brains, while both oxidized and unoxidized PRDX2 were identified in the control brains [33].

In contrary to PRDX1 and PRDX2, PRDX3 is downregulated in the AD brain tissues compared to controls [18,31]. Loss of PRDX3 has also been reported in transgenic mice expressing human APP, a model of AD [41]. Overexpression of PRDX3 appeared to have a protective role against (PQ)-induced mitochondrial damage in mice cerebral cortex. PQ-exposed transgenic PRDX3 mice displayed significantly lower level of Aβ than PQ-exposed wild type animals. Moreover, PQ-exposed APP transgenic mice with the PRDX3 transgene (APP/PRDX3) had significantly decreased levels of Aβ compared to the PQ-exposed APP transgenic mice without the PRDX3 transgene (APP/WT), suggesting that overexpression of PRDX3 suppressed PQ-induced amyloidogenesis. In addition, learning and memory impairment induced by PQ was significantly attenuated by PRDX3 overexpression in APP transgenic mice [42]. In the recent study, the same group of authors has also shown that PRDX3 overexpression improved cognitive ability of APP transgenic mice and reduced mitochondrial oxidative stress in these animals [43].

Current knowledge regarding the role of PRDX4 in PRDX5 in AD is limited to some experimental data. Thus, in HEK293T cells, simultaneous silencing of APP and its family members, APLP1 and APLP2, diminished the level of PRDX4 protein, but not mRNA [44]. Of note, PRDX4 was localized in endoplasmic reticulum (ER) in HEK293T cells [44]. Recently, Kam and co-workers showed that treatment of mouse hippocampal neurons HT-22 with Aβ oligomer (AβO) induced ER stress and changed ER morphology, which in turn increased the PRDX4 level. Furthermore, PRDX4 overexpression decreased, and PRDX4 silencing increased AβO-mediated ROS production and ER stress. AβO-induced ER stress resulted in elevation of intracellular Ca^2+^ which was prevented by PRDX4 overexpression. Finally, PRDX4 silencing enhanced and PRDX4 overexpression attenuated AβO-triggered apoptosis in HT-22, strongly suggesting a protective role of PRDX4 in ameliorating AβO-induced ER stress and neuronal cell death [45]. In the latest study, the same authors documented induction of PRDX4 expression in HT-22 cells treated with glutamate (Glu), a neurotoxin contributing to neurodegeneration. PRDX4 overexpression diminished Glu-induced apoptosis by inhibition of ROS production, Ca^2+^ influx and ER stress, further underscoring a neuroprotective role of PRDX4 [46].

Similarly to PRDX4, also PRDX5 protects HT-22 cells against Glu-induced toxicity. The expression of PRDX5 is elevated in Glu-treated cells and silencing of PRDX5 augments Glu-induced apoptosis [47]. Likewise, a neuroprotective role of PRDX5 has been documented in HT-22 cell treated with AβO. A significant increase in the level of PRDX5 was observed in both compartments in neuronal cells exposed to AβO. Transfection with PRDX5 decreased AβO-induced generation of ROS and peroxynitrite, which in turn inhibited ERK-Drp1-mediated synuclein mitochondrial fragmentation and neuronal cell death. Conversely, knockdown of PRDX5 increased AβO-induced generation of ROS and cell death [48]. The same group of authors documented neuroprotective function of PRDX5 in the other cellular model of AD as well as in animal model of this disease. Additionally, they demonstrated that induction of PRDX5 by AβO modulates Ca^2+^-mediated activation of calpain, a key regulator of p35 cleavage to p25. Inhibition of conversion of p35 to p25 decreases Cdk5 activity which in turn prevents cell death. Collectively, those results suggest that PRDX5 may inhibit AβO-induced neurodegeneration via preventing Cdk5 activation [49].

It is also worth mentioning that recent studies point to the role of PRDX5 in neuronal cell death induced by iron overload, a phenomenon observed in several neurodegenerative diseases, including AD. Thus, an exposure of HT-22 cells to iron induces ER stress and mitochondrial fragmentation, reduces the phosphorylation level of Drp1, a key mitochondrial fission protein, as well as significantly increases the PRDX5 level. Silencing of PRDX5 aggravates, while PRDX5 overexpression attenuates iron overload-induced mitochondrial damage and maintains phosphorylation of Drp1 at control level. Furthermore, PRDX5 overexpression inhibits, while PRDX5 silencing aggravates apoptosis in HT-22 cells exposed to iron, indicating that this protein prevents iron overload-induced neuronal death [50]. Recently, this neuroprotective role of PRDX5 has also been documented in iron-loaded mice. Knockdown of PRDX5 aggravates hippocampal iron accumulation as well as exacerbates iron overload-induced ER stress, Drp1-mediated mitochondrial fission and neuronal death in mice hippocampi. Moreover, ROS levels in mice fed a high iron diet are significantly higher in PRDX5 knockouts compared to WT animals. Treatment with N-acetyl-cysteine (NAC), a ROS scavenger, inhibits ROS production as well as ER stress, mitochondrial fission and neuronal loss in PRDX5 deficient mice, strongly indicating that lack of PRDX5 aggravates iron overload-induced neuronal death by increasing ROS production [51].

Of note, PRDX5 displays not only a neuroprotective, but also an anti-inflammatory activity. ROS-dependent induction of PRDX5 expression has been detected in LPS-stimulated microglia. Overexpression of PRDX5 ameliorated the production of pro-inflammatory mediators, TNF-a, IL-1β, and IL-6. Moreover, PRDX5 prevented LPS-induced mitochondrial fission through inhibition of activity of ROS-dependent Ca_2+_/calcineurin and Drp1 [52]. Given the involvement of microglia in neurogenesis and neuroinflammation, these findings underscore the role of PRDX5 in the prevention of neurodegeneration process.

There are inconsistent data on expression of PRDX6 in AD tissue. Krapfenbauer and co-workers did not observed any changes in the level of PRDX6 between AD and control tissues [32]. Contrary to this, Power and colleagues found a marked increase in the level of PRDX6 in astrocytes, but not neurons, in both gray and white matter in AD tissue compared to the control tissue. The same study revealed that PRDX6 positive astrocytes and their projections were localized in close proximity to plaques containing aggregations of Aβ. Of note, strong PRDX6 staining was observed in the walls of many blood vessels which had multiple contacts with PRDX6 positive activated astrocytes [27].

Yun and co-workers have shown that overexpression of PRDX6 promotes amyloidogenesis in the brains of Aβ1-42-infused mice compared to the wild type animals infused with Aβ1-42 [53]. Moreover, Aβ1-42-induced memory impairment in PRDX6 transgenic mice was more pronounced than in the wild-type mice. Furthermore, astrocytes and microglia of Aβ1-42-infused PRDX6 transgenic animals were more activated compared to astrocytes and microglia of the wild-type mice. Aβ1-42 infusions resulted in the increase of PRDX6 level and the PLA2 activity. The authors suggested that the elevated PLA2 activity could significantly contribute to the worse status of AD in PRDX6 transgenic mice [53]. More recently, the same group have shown that PRDX6 levels were increased by an antioxidant thiacremonone to prevent Aβ1-42/H_2_O_2_-induced oxidative stress in cultured neuronal cells and the brains of APP/presenilin 1 (PS1) mice. Based on these findings the authors hypothesized that PRDX6 could contribute to the beneficial effects of thiacremonone [54].

A very recent study clearly shows that PRDX6 overexpression reduces Aβ load in the mice brain cortex and hippocampus, while PRDX6 haplodeficiency increases it. PRDX6 overexpression did not affect hyperactive astrogliosis. PRDX6 haplodeficiency did not impair ability of astrocytes to mount response to Aβ deposition. Interestingly, activation of microglia varied directly with the PRDX6 gene dose. Phagocytic activation of plaque-associated microglia was enhanced in mice overexpressing PRDX6 and attenuated in PRDX6 haplodeficient mice. PRDX6 protein was exclusively expressed by astrocytes but undetectable in plaque-associated microglia. Finally, PRDX6 overexpression inhibited and haplodeficiency promoted seeding of Aβ plaque. In light of these results, it is tempting to speculate that astrocytes rather than being passive by-standers actively recognize Aβ deposits and modulate microglia phagocytic activity to counter Aβ accumulation. PRDX6 mediated resistance to oxidative stress allows astrocytes to penetrate plaques that escape initial seeding control. Therefore, upregulation of PRDX6 expression may be a promising tool for AD treatment [55].

The effects of manipulation of expression of genes encoding particular PRDXs in AD models are summarized in Table 2.

### 4.2. PRDXs in Parkinson’s Disease (PD)

PD is the most common neurodegenerative disorder that affects movement. In 2016, 6.1 million individuals had PD globally [56]. The main PD symptoms include, but are not limited to: tremor, rigidity, bradykinesia/akinesia and postural instability. The first symptoms may be barely noticeable and gradually worsen over time. The hallmarks of PD are the loss of dopaminergic neurons and the presence of Lewy bodies (LBs). LBs are intraneuronal inclusions composed of more than 90 proteins, of which the most abundant are α-synuclein (a-syn) and ubiquitin. α-syn has the propensity to misfold and accumulate as insoluble intracellular inclusions. Aside from SNCA, coding for a-syn, several other genes encoding parkin (PRKN), PTEN-induced putative kinase I (PINK1), DJ-1 protein (DJ-1), leucine-rich repeat serine/threonine-protein kinase 2 (LRRK2) and glucocerebrosidase (GBA) have been linked to PD. Parkin, PINK1 and DJ-1 regulate mitochondrial functions. LRRK2 interferes with autophagy and GBA is a lysosomal enzyme which metabolizes glucosylceramide. Parkinsonism is present in 85% of patients suffering from dementia with Lewy bodies (DLB), a progressive dementia in which cognitive impairment begins simultaneously or within 1 year of parkinsonism. Similarly to PD, DLB is characterized by the presence of LBs and neuronal loss [57].

The expression level of PRDX1 in the brain tissues from patients with PD has not been examined so far. However, there is some evidence suggesting that this protein may exert a protective role in experimental models of this disease. Treatment of dopaminergic neuronal cells and primary cultures of mesencephalic neurons with 6-hydroxydopamine (6-OHDA) induced cell death and generation of ROS, which in turn triggered oxidative modification of PRDX1. These findings were confirmed in the brains of 6-OHDA-lesioned rats. Moreover, overexpression of PRDX1 protected neuronal cells against death induced by 6-OHDA by scavenging ROS [58].

Treatment of mice with 6-OHDA decreased the acetylation levels of PRDX1 and PRDX2. An inhibitor of HDAC6, tubastatin A, significantly attenuated 6-OHDA induced deacetylation of either protein and reduced ROS production. Therefore, accumulation of acetylated PRDX1 and PRDX2 may contribute to the neuroprotective properties exerted by tubastatin A [59].

In a very recent study, Wirakiat and co-workers analyzed the relationship between PRDX1 and a transcription factor eEF1A2 during neuronal differentiation and in SH-SY5Y cells treated with a neurotoxin 1-methyl-4-phenylpyridinium (MPP^+^), a model of PD. The protein, but not mRNA, level of PRDX1 decreased during differentiation. Upon MPP^+^ treatment, the level of PRDX1 decreased in the undifferentiated and increased in differentiated cells. Treatment with H7, a PRDX1 inhibitor [60], decreased the viability of differentiated SH-SY5Y cells and treatment with both H7 and MPP^+^ resulted in further reduction of viability. Moreover, treatment with either H7 or MPP^+^ triggered apoptosis, which was enhanced upon co-treatment with both compounds. Treatment with H7 and MPP^+^, alone or in combination, induced ROS production as well as elevated the levels of p53 and phosphorylated forms of AKT and mTOR. In MPP^+^-treated cells PRDX1 colocalized with eEF1A2 and this colocalization was not observed when the cells were co-treated with H7, suggesting that disruption of the eEF1A2 and PRDX1 interaction may enhance cell death in the neurons subjected to H7 and MPP^+^ co-treatment [61].

An increased level of PRDX2 has been documented in the brain tissues form patients with PD compared to controls [62]. Overexpression of PRDX2 significantly attenuated 6-OHDA neurotoxicity in both in vitro and in vivo settings. Moreover, PRDX2 presented antiapoptotic effects in 6-OHDA-treated neurons by inhibiting caspase-dependent apoptosis and suppression of apoptosis signal-regulating kinase (ASK1) signaling cascade [63].

The activity of PRDX2 is reduced through phosphorylation by Cdk5. A significantly elevated level of phospho-PRDX2 was observed in cultured mouse cortical neurons exposed to MPP^+^. Overexpression of PRDX2 protected neurons from death after MPP^+^ insult, while downregulation of PRDX2 increased oxidative stress resulting in neuronal death. Moreover, PRDX2 overexpression prevented the loss of dopaminergic neurons in MPTP mouse model of PD. A significant increase in phospho-PRDX2 positive neurons was observed in PD patient tissues compared to controls supporting the importance of PRDX2 in this disease [64].

PRDX2 enzymatic activity and protective function form oxidative stress are also inhibited by S-nitrosylation. Significantly increased levels of S-nitrosylated PRDX2 (SNO-PRDX2) were found in the brain tissues from PD patients compared to controls [65] and in models of both murine and human PD [65,66]. Transfection with sulfiredoxin (Srxn1), an enzyme denitrosylating PRDX2, abolished S-nitrosylation of PRDX2 in dopaminergic neurons and significantly decreased the number of apoptotic neurons after exposure to PQ and maneb (MB), toxins linked epidemiologically to PD [66].

An elevation of phosphorylated PRDX3 has been found in human PD brain tissues compared to normal brain tissues and this increase was most pronounced in the PD tissues expressing mutant leucine-rich repeat kinase 2 (LRRK2) which is linked to autosomal dominant PD. LRKK2 mutations elevated PRDX3 phosphorylation, which in turn decreased peroxidase activity of this protein. Overexpression of mutated LRKK2 in neuronal cells induced apoptosis, ROS production, molecular damage and mitochondrial dysfunction. These damaging effects were exacerbated in PRDX3-depleted cells implying rescue by PRDX3 from LRKK2-induced oxidative stress [67].

Silencing of PRDX5 in SH-SY5Y neuroblastoma cells increased vulnerability to oxidative damages induced by MPP^+^ [68]. Transfection of SH-SY5Y cells with PRDX5 prevented caspase-dependent apoptosis and mitochondrial DNA damage induced by MPP^+^. Moreover, overexpression of PRDX5 abolished an increase in Ca^2+^ and calpain activity caused by MPP^+^, suggesting that this enzyme acts upstream of calpain stimulation by Ca^2+^ and therefore may control release of Ca^2+^ [69].

A very recent study documented a decrease of PRDX5 expression in both cellular and rat rotenone-induced PD model. Silencing of PRDX5 sensitized neurons to apoptosis triggered by rotenone treatment and enhanced activation of the ATM/p53/PUMA apoptotic pathway. PRDX5-silenced cells displayed the increased levels of intracellular ROS and mitochondrial superoxide compared to the control cells. Interestingly, PRDX5 silencing-evoked DNA damage manifested as the γ-H2AX expression was not attributed to the higher levels of ROS [70].

The significantly elevated levels of PRDX6 has been documented in the PD and dementia with Lewy bodies (DLB) brain tissues compared to the control brains [71]. There were 10 to 15 more PRDX6-positive cells in gray matter and 3-times more positive cells in white matter than in control cortices. Most of the PRDX6-positive astrocytes were larger with many processes indicating hyperplastic changes. Some neurons in the PD and DLB brain tissues were positive for both PRDX6 and α-synuclein, the main component of Lewy bodies, and a direct interaction between α-synuclein and PRDX6 was documented [71]. Genetic depletion of 26S proteasomes in mouse brain neurons causes neurodegeneration and the Lewy-like inclusion body formation, resembling features apparent in the brains of patients with PD and DLB [72]. The amount of PRDX6, PLA2 activity and the level of lipid peroxidation are significantly increased in the 26S proteasome-depleted versus control mice cortex. Moreover, a much higher diffuse PRDX6 staining has been found in the 26S proteasome-depleted cortical brain sections compared to the control, which suggests that this protein could be secreted by activated astrocytes. Additionally, an inverse relationship has been found between the levels of PRDX6 protein expression and ROS in 26S proteasome-depleted mouse cortex with increasing age, which might suggest a neuroprotective function of PRDX6 in response to neurodegeneration caused by 26S proteasome depletion [73].

However, the role of PRDX6 in PD, as in the other neurodegenerative disorders, seems to be controversial. Yun and co-workers analyzed the influence of PRDX6 on MPTP-induced dopaminergic neurodegeneration using PRDX6 Tg mice. The behavioral deficit evoked by MPTP administration was more evident in PRDX6 Tg mice compared to WT animals. Moreover, damage of dopaminergic neurons and ROS production was higher in PRDX6 Tg mice than in WT mice. An increased release of TNF-α, IL-1β and neurotoxic 4-hydroxynonenal (4-NHE), induced by treatment with MPP^+^, was observed in astrocytes isolated from PRDX6 Tg mice compared to those isolated from WT animals. These results suggest that PRDX6 induces astrogliosis and astrocytes-mediated neurodegeneration [74]. More recently, the same group of authors showed that neural stem cells from PRDX6 Tg mice resisted spontaneous differentiation for a prolonged time and were unsuccessful in forming a high-quality network. PRDX6 inhibited the neurogenesis of neural precursor cells through toll-like receptor 4 (TLR4)-dependent downregulation of WD-repeat- and FYVE-domain-containing protein 1 (WDFY1) [75]. Collectively, these results suggest an inhibitory effect of PRDX6 on neurogenesis which may contribute to the development of neurodegenerative diseases.

The outcomes of manipulation of expression of genes coding for particular PRDXs in PD models are summarized in Table 3.

### 4.3. PRDXs in Multiple Sclerosis (MS)

MS is the most frequent chronic inflammatory disease of the CNS which affects more than 2 million people worldwide. It is characterized by fully or partially reversible episodes of worsening of neurological functions which may last for days or weeks. Typical symptoms include monocular visual loss, double vision, limb weakness, sensory loss, and ataxia. Over time, many patients develop a progressive mobility and cognition impairment. MS is characterized by destruction of myelin, inflammation, and glial reaction. It is still unclear whether the root cause of tissue damage observed in MS is intrinsic to the CNS or extrinsic. Nevertheless, several studies indicate a crucial role of adaptive immunity in MS pathogenesis [76]. Inflammation of the white and gray matter resulting from infiltration of immune cells, mainly T helper cells and B lymphocytes, and their cytokines seems to be an incipient cause of damage in MS [77].

Enhanced vascular PRDX1 immunoreactivity was observed in the active demyelinated MS lesions containing numerous macrophages, while in the control brain tissue and normal appearing white matter this protein was mainly detected in glial cells and only its traces were found in the vasculature. In brain specimens from animals with experimental autoimmune encephalomyelitis (EAE), a model of MS, PRDX1 expression was observed in glial cells and a weak PRDX1 signal was found in blood vessels without perivascular infiltrates. The level of this protein was increased in brain endothelial cells (BECs) surrounded by inflammatory cells. Overexpression of PRDX1 protected BECs from ROS-induced cell death, reduced adhesion and transendothelial migration of monocytes by downregulation of intracellular adhesion molecule-1 and enhanced the integrity of the BEC layer [78].

PRDX2 was upregulated in white matter astrocytes in tissues form MS patients compared to control material. A slight PRDX2 signal was also observed in activated microglia/macrophages in MS tissues. A strong positive correlation was found between the number of astrocytes expressing PRDX2, T cell infiltration and the activation of microglia/macrophages, two markers of inflammation. Furthermore, PRDX2 level in MS lesions was also positively correlated with the expression of NQO1, an indicator of oxidative stress [23].

Similarly to PRDX1 and PRDX2, also PRDX3 appeared to be upregulated in MS lesions compared to surrounding normal appearing white matter and this elevation was more pronounced in the early active lesions than in late active lesions. In early active lesions expression of PRDX3 was localized to astrocytes and oligodendrocytes, while in late active lesions it was absent in oligodendrocytes. In the same study, overexpression of PRDX3 in astrocytoma cells reduced ROS production and increased viability of astrocytes and surrounding neurons upon treatment with H_2_O_2_ [79].

Holley and co-workers analyzed the expression of PRDX5 in white matter from human brain and MS patients. An increase in PRDX5 level was found in MS normal-appearing white matter, but it was more pronounced in astrocytes in MS lesions. PRDX5 immunostaining was observed in hypertrophic astrocytes in acute lesions displaying inflammation, but also in post-reactive astrocytes in chronic lesions with abated inflammation which could suggest ongoing oxidative stress despite the absence of histologically defined inflammation [80].

A strong expression of PRDX6 was found in MS patient and in EAE animals. In EAE animals, PRDX6 was detected in astrocytes, while lack of this protein was observed in neurons and oligodendrocytes. The increased PRDX6 level in EAE mice reduced expression of matrix metallopeptidase 9 (MMP9) and fibrinogen leakage, diminished immune cell infiltration, microglia activation and prevent blood-brain-barrier (BBB) disruption, leading to protection from the damage of the spinal cord by EAE. Although PRDX6 transgenic mice exhibited neurological signs of EAE starting at the same time as wild type mice, they were significantly less severe. Moreover, the myelin loss was inhibited in PRDX6 transgenic mice compared to wild type animals, indicating that PRDX6 prevented demyelination by EAE in the spinal cord [81].

In a very recent study, Uzawa at co-workers analyzed for the first time the amount of PRDX1, PRDX5 and PRDX6 in CSF and serum of patients with MS, neuromyelitis optica spectrum disorder (NMOSD), and other neurological disorders (ONDs). The mean values (ng/mL) of PRDXs in CSF varied between 1.17 and 3.94 ng/mL no significant differences in the amount of PRDXs were found among patients with NMOSD, MS and ONDs. The amounts of these proteins in serum varied between 0.98 and 43.81 (ng/mL). The amounts of PRDX5 and PRDX6 in patients with MS and NMOSD were significantly higher than those in patients with ONDs [82].

The effects of manipulation of expression of genes encoding particular PRDXs in MS models are summarized in Table 4.

### 4.4. PRDXs in Amyotrophic Lateral Sclerosis (ALS)

ALS was originally defined as a pure motor neuron disease, but is now recognized as a multisystem neurodegenerative disorder. ALS is included to a category of very rare motor neuron diseases (MNDs) [83]. In 2016, there were around 400,000 cases of MND across the world. The clinical presentation of ALS typical consists of muscle weakness, accompanied by muscle atrophy, slowness of movements with muscle stiffness. The disease is progressive in most patients, and death is mostly attributed to respiratory failure. ALS is characterized by loss of the neuromuscular connection, axonal retraction and subsequent cell death of motor neurons, accompanied by astro- and microgliosis. Protein aggregation, excitotoxicity, neuroinflammation, mitochondrial dysfunction and oxidative stress, cytoskeletal disturbances, and disturbed RNA processing are thought to be implicated in pathogenesis of ALS. Among an increasing list of genes recognized as associated with ALS, the most commonly described are: C9orf72, SOD1, TARDBP, and FUS [84].

In patients with sporadic ALS (SALS) PRDX2 and GPx1 were strongly co-expressed in residual neurons within about 3-years after disease onset, and a number of neurons expressing both proteins decreased with disease progression. In patients with familial ALS (FALS) with SOD1 mutations co-expression of PRDX2, GPx1 and SOD1 was found in Lewy body-like hyaline inclusions (LBHIs), but certain residual motor neurons without inclusions also overexpressed PRDX2 and GPx1. In the ALS animal models certain residual motor neurons showed overexpression of PRDX2 and GPx1 which was disrupted at the terminal stage of ALS. These findings suggested that the residual ALS neurons displaying upregulation of redox system might be less susceptible to ALS stress and protect themselves from ALS neuronal death. The breakdown of this redox system observed at the advanced disease stage might potentiate neurodegeneration and neuronal loss [85].

Downregulation of PRDX3 has been detected in the presence of mutant SOD1G93A motor neuronal-like NSC34 cells, in the spinal cord mitochondria of mutant SOD1 transgenic mice, but also in spinal motor neurons from patients with both sporadic and SOD1-related motor neuron disease (MND). Interestingly, the same study has also revealed the reduction of PRDX4 and an increased level of PRDX2 in mutant SOD1G93A neuronal cells. Ebselen, an anti-oxidant drug mimicking PRDX activity, ameliorated the toxicity of mutant SOD1 in these cells [25]. Overexpression of PRDX3 significantly increased survival and reduced oxidative stress in mutant SOD1G93A NSC34 cells [86].

Contrary to above presented studies, Pharaoh and co-workers observed increased levels of PRDX2, PRDX3, and PRDX6 in spinal cords from SOD1G93A mice compared to wild-type control [87]. Interestingly, upregulation of PRDX6 was detected in spinal cords of paralyzed SOD1G93A mice compared to the controls, while it was not found in asymptomatic 6- to 8-week-old SOD1G93A mice, suggesting that PRDX6 may be involved in the later stages of the disease [88]. Further studies are needed to elucidate whether this discrepancy in the detected in SOD1G93A model level of PRDX3 described in different studies reflects the stage of ALS.

### 4.5. PRDXs in Huntington’s Disease (HD)

HD is a rare hereditary neurodegenerative disorder characterized by cognitive, behavioral, and motor symptoms which lead to increasing disability and functional decline. The global population prevalence of HD varies across regions from 0.02/100 000 in South Africa to 12.3/100 000 in the UK [89,90]. HD is an autosomal dominant single-gene disorder. The mutation is an expanded CAG triplet repeat near the exon 1 of the Huntingtin gene (HTT), which leads to the presence of a polyglutamine (polyQ) stretch at the N-terminus of Huntingtin protein (HTT). Mutated HTT (mHTT) is implicated in several mechanisms which include, among others, intracellular inclusions of mHTT, transcriptional dysregulation, mitochondrial dysfunction, excitotoxicity, and inflammation. Ultimately there is massive striatal degeneration in the patient brain [90].

An elevated level of PRDX1, PRDX2 and PRDX6 was documented in the striatal samples of HD compared to controls, while PRDX3 and PRDX5 showed no differences in expression [91]. Moreover, PRDX1 was also upregulated in induced pluripotent stem cell (iPSC) lines derived from HD patients compared to normal iPSCs and human embryonic stem cells (hESCs) [92]. Transfection of PC12 cell with a sequence encoding mutant huntingtin (mHtt), a key component of HD, decreased the level of PRDX1. The same study revealed that overexpression of PRDX1 attenuates and its knockdown potentiates mHtt-induced toxicity in PC12 cells [93].

In a recent study, Agrawal and Fox performed proteomic analysis of mitochondria isolated from brains of 12 weeks old R6/2 HD or 15 months old YAC128 HD mice. R6/2 HD mice express the exon-1 fragment of the human HTT transgene and have advanced disease at 12 weeks of age. YAC128 HD mice express full-length human mHtt with 128 glutamine repeats and at 15 months of age are considered as a model of middle-stage HD. A decrease in the PRDX3 level was detected in both models, which was consistent with decreased peroxide and peroxynitrite scavenging capacity. A diminished level of PRDX2 was found in R6/2 HD suggesting further decreases in peroxide scavenging with advancing HD [94].

## 5. Conclusions

Deregulated expression and/or activity of PRDXs has been observed in various neurodegenerative diseases (Table 5) as well as their experimental models (Table 6), but clearly further in-depth analyses are required to obtain a comprehensive picture of these alterations. Whether and how the particular alterations contribute to the pathogenesis of neurodegenerative disorders remains unclear. An elevated level of PRDXs may represent a defensive compensatory reaction to the oxidative damage, as several studies indicate that these enzymes protect neurons against oxidative stress via detoxification of ROS. Increasing evidence suggests also that the role of PRDXs may go beyond an antioxidant properties and could be related to the regulation of cell signaling. On the other hand, diminished level of PRDXs documented in some studies may be a consequence of mitochondrial damage shown in certain neurodegeneration conditions. Restoration of redox balance may play an important role in minimizing the detrimental effects of oxidative damage in neurodegenerative disorders. Whether modulation of the activity of PRDXs may offer a useful approach to re-establish redox homeostasis is still an open question.

## Figures and Tables

**Table 1:** PRDXs expression in the central nervous system (CNS).

	PRDX1	PRDX2	PRDX3	PRDX4	PRDX5	PRDX6
neurons	− [18]; +/− [19,20,21,22]	+ [18]	+ [19,20,24,25]	+ [19]	+ [19,20]	+/− [19,27]
astrocytes	+ [18,22]	+ [18]	+ [26]	+ [19]	n/a	+ [19,27]
oligodendrocytes	+ [19,20,21,22]	n/a	n/a	+ [20]	n/a	+ [20,26]
ependymocytes	+ [18,22]	n/a	n/a	n/a	n/a	n/a
microglia	+ [19,20,21]	n/a	n/a	n/a	n/a	n/a

+, expression; −, lack of expression; +/−, minimal expression; n/a, not available.

**Table 2:** The influence of modulation of *PRDXs* expression on phenotype of Alzheimer’s disease (AD) models.

Treatment	Model	Outcome	Ref.
*PRDX1* overexpression	PC12 cells	protection against Aβ toxicity	[33]
	SH-SY5Y cells	protection against Aβ toxicity; decreased pro-BDNF; increased TrkB and pERK5	[35]
*PRDX2* overexpression	cortical neurons	protection against Aβ toxicity	[40]
*PRDX3* overexpression	APP transgenic mice	protection against PQ toxicity; suppressed PQ-induced amyloidogenesis; improvement of cognitive ability	[42,43]
*PRDX4* overexpression	HT-22 neurons	decreased AβO-mediated ROS production, ER stress and apoptosis	[45]
		decreased Glu-induced apoptosis, ROS production, Ca^2+^ influx and ER stress	[46]
*PRDX4* silencing	HT-22 neurons	increased AβO-mediated ROS production, ER stress and apoptosis	[45]
*PRDX5* overexpression	HT-22 neurons	decreased AβO-induced production of ROS, mitochondrial fragmentation and apoptosis	[48]
		decreased iron overload-induced mitochondrial damage and apoptosis	[50]
	N2a-APPswe cells	protection against Aβ toxicity; decreased ROS production	[49]
	microglia	decreased production of LPS-induced pro-inflammatory mediators, mitochondrial fission	[52]
*PRDX5* knockdown	HT-22 neurons	increased AβO-induced generation of ROS and apoptosis	[48]
		increased iron overload-induced mitochondrial damage and apoptosis	[50]
	C57BL/6 mice	increased iron overload-induced ROS production, ER stress, mitochondrial fission and neuronal death	[51]
*PRDX6* overexpression	C57BL/6 mice	accelerated Aβ-induced memory decline	[53]
		decreased Aβ load; increased activation of microglia	[55]
*PRDX6* haplodeficiency	C57BL/6 mice	increased Aβ load; attenuated activation of microglia	[55]

**Table 3:** The influence of modulation of *PRDXs* expression on phenotype of PD models.

Treatment	Model	Outcome	Ref.
*PRDX1* overexpression	MN9D DA neuronal cells	protection against 6-OHDA toxicity	[58]
PRDX1 inhibition with H7	SH-SY5Y cells	decreased cell viability; induced ROS production and apoptosis	[61]
*PRDX2* overexpression	MN9D DA neuronal cells; C57BL/6 mice	protection against 6-OHDA toxicity	[63]
	primary mice neurons	protection against MPP^+^ toxicity	[64]
	C57BL/6 mice	decreased MPTP-induced loss of neurons	
*PRDX5* overexpression	SH-SY5Y cells	decreased apoptosis and mitochondrial DNA damage induced by MPP^+^	[69]
*PRDX5* silencing	SH-SY5Y cells	increased vulnerability to oxidative damages induced by MPP^+^	[68]
		sensitization to rotenone-induced apoptosis	[70]
*PRDX6* overexpression	C57BL/6 mice	accelerated behavioral deficit evoked by MPTP; increased damage of neurons and ROS production	[74]

**Table 4:** The influence of modulation of *PRDXs* expression on phenotype of multiple sclerosis (MS) models.

Treatment	Model	Outcome	Ref.
*PRDX1* overexpression	rat brain endothelial cells	decreased ROS-induced death; increased blood-brain-barrier integrity	[78]
*PRDX3* overexpression	U373 astrocytoma cells	reduced ROS production; increased viability of astrocytes and surrounding neurons upon treatment with H_2_O_2_	[79]
*PRDX6* overexpression	C57BL/6 mice	suppressed severity of EAE; decreased weight loss; reduction in blood-brain-barrier disruption, peripheral immune cell infiltration, and neuroinflammation	[81]

**Table 5:** *PRDXs* expression in the brains of patients with neurodegenerative diseases.

	AD	PD	MS	ALS	HD
PRDX1	up [31,33,34]; unch. [32]	*n*/a	up [78]	*n*/a	up [91]
PRDX2	up [31,32,34,40]	up [62]	up [23]	up [85]	up [91]
PRDX3	down [18,31]	up [67]	up [79]	*n*/a	up [91]
PRDX4	*n*/a	*n*/a	*n*/a	*n*/a	*n*/a
PRDX5	*n*/a	*n*/a	up [80]	*n*/a	unch. [91]
PRDX6	unch. [32]; up [27]	up [71]	up [81]	*n*/a	up [91]

up, upregulation; down, downregulation; unch., unchanged expression compared to the controls; *n*/a, not available.

**Table 6:** *PRDXs* expression in the experimental models of neurodegenerative diseases.

	AD	PD	MS	ALS	HD
PRDX1	up in Aβ-resistant PC12 cells [33]	decreased level of acetylated form in 6-OHDA-treated mice [59]; downregulated in MPP^+^-treated SH-SY5Y cells [60]	up in mice with EAE [78]	n/a	n/a
PRDX2	up in mice expressing ABAD and mutated APP [40]	decreased level of acetylated form in 6-OHDA-treated mice [59]; increased level of phosphorylated form in neurons exposed to MPP^+^ [64]; decreased S-nitrolysation in neurons exposed to PQ and MB [66]	n/a	up in mice with SOD1 mutation [85,87]; up in SOD1G93A NSC34 cells [25]	down in R6/2 mice [94]
PRDX3	down in mice expressing APP [41]	n/a	n/a	down in SOD1G93A NSC34 cells, and in mutant SOD1 mice [25]; up in SOD1G93A mice [87]	down in R6/2 and YAC128 mice [94]
PRDX4	down in HEK293T cells with silenced APP, APLP1 and APLP2 [44]; up in HT-22 cells treated with AβO [45] or Glu [46];	n/a	n/a	down in SOD1G93A NSC34 cells [25]	*n*/a
PRDX5	up in HT-22 cells treated with Glu [47] or AβO [48]	down in cellular and rat rotenone-induced PD models [70]	n/a	n/a	n/a
PRDX6	up in Aβ1-42 infused mice [53]	n/a	up in mice with EAE [81]	up in SOD1G93A mice [87,88]	n/a

up, upregulation; down, downregulation; n/a, not available.
